# The energy metabolism of *Balantidium polyvacuolum* inhabiting the hindgut of *Xenocypris davidi*

**DOI:** 10.1186/s12864-023-09706-6

**Published:** 2023-10-19

**Authors:** Xia-lian Bu, Wei-shan Zhao, Zhong-yang Li, Hong-wei Ma, Yu-shun Chen, Wen-xiang Li, Hong Zou, Ming Li, Gui-tang Wang

**Affiliations:** 1 State Key Laboratory of Freshwater Ecology and Biotechnology, Key Laboratory of Aquaculture Disease Control, Ministry of Agriculture, Institute of Hydrobiology, Chinese Academy of Sciences, Wuhan, 430072 Hubei China; 2 University of Chinese Academy of Sciences, Beijing, 100049 China; 3 College of Veterinary Medicine, Huazhong Agricultural University, Wuhan, 430070 Hubei China

**Keywords:** *Balantidium polyvacuolum*, Anaerobic ciliate, Endosymbiont, Mitochondrion-related organelles (MROs)

## Abstract

Anaerobic parasitic ciliates are a specialized group of ciliates that are adapted to anoxic and oxygen-depleted habitats. Among them, *Balantidium polyvacuolum*, which inhabits the hindgut of Xenocyprinae fishes, has received very limited scientific attention, so the molecular mechanism of its adaptation to the digestive tract microenvironment is still unclear. In this study, transmission electron microscopy (TEM) and single-cell transcriptome analysis were used to uncover the metabolism of *B. polyvacuolum*. Starch granules, endosymbiotic bacteria, and multiple specialized mitochondrion-related organelles (MROs) of various shapes were observed. The MROs may have completely lost the electron transport chain (ETC) complexes I, III, IV, and V and only retained succinate dehydrogenase subunit A (SDHA) of complex II. The tricarboxylic acid (TCA) cycle was also incomplete. It can be inferred that the hypoxic intestinal environment has led to the specialization of the mitochondria in *B. polyvacuolum*. Moreover, carbohydrate-active enzymes (CAZymes), including carbohydrate esterases, enzymes with a carbohydrate-binding module, glycoside hydrolases, and glycosyltransferases, were identified, which may constitute evidence that *B. polyvacuolum* is able to digest carbohydrates and starch. These findings can improve our knowledge of the energy metabolism and adaptive mechanisms of *B. polyvacuolum*.

**Supplementary Information:**

The online version contains supplementary material available at 10.1186/s12864-023-09706-6.

## Introduction

Ciliates (protozoa of the phylum Ciliophora) are single-celled organisms characterized by motile cilia, nuclear dimorphism and conjugation [1]. Members of this group are very diverse and can inhabit a wide variety of environments. Species of *Balantidium* Claparède & Lachmann, 1858 have been widely found in the digestive tracts of animals, including amphibians, fishes, pigs, and humans [2–4]. *B. polyvacuolum* is an endocommensal found in the hindgut of Xenocyprinae fishes [5, 6], whose living environment is almost anaerobic. Only a few studies about this species are available, and they focus on its morphology and phylogeny [5–7]. No genomic data are available for this ciliate species.

Mitochondria are essential organelles in eukaryotic life forms that have diverse forms and functions in different environments and evolve under different selective pressures [8, 9]. Previous studies have shown that anaerobic ciliates such as *Cyclidium porcatum*, *Metopus contortus*, and *Plagiopyla frontata* have evolved specialized mitochondria, called hydrogenosomes, that can produce hydrogen and adenosine triphosphate (ATP) [10–14]. Anaerobic ciliates with hydrogenosomes typically harbor endosymbiotic bacteria and archaea, which may establish intricate interactions with the hydrogenosomes in some cases [14–16]. These bacteria and archaea use the supplied hydrogen to convert energy; in return, ciliates can avoid the pressure of hydrogen [17].

The hydrogenosomes of *B. polyvacuolum* and its endosymbionts remain poorly understood. In this study, we therefore investigated the energy metabolism of *B. polyvacuolum* based on single-cell transcriptome data. Cluster of Orthologous Group (COG) annotation, Kyoto Encyclopedia of Genes and Genomes (KEGG) pathway annotation, and carbohydrate-active enzyme (CAZyme) identification were conducted. In addition, the ultrastructural morphology of hydrogenosomes and the endosymbiont bacteria was also revealed with the aid of transmission electron microscopy (TEM).

## Materials and methods

### Specimen collection

The hosts (n = 9) of *B. polyvacuolum*, *Xenocypris davidi*, were collected from the Yangtze River in Xianning City, Hubei Province, China in July 2022 and transported alive to the laboratory for further examination. Fishes were anesthetized using 0.02% tricaine methane sulfonate (MS-222, Sigma) according to the manufacturer’s protocol and dissected in accordance with the protocols approved by the Animal Ethics Committee of Institute of Hydrobiology, Chinese Academy of Sciences (IHB/LL/2,023,036). The luminal contents of the fish intestines were transferred into Petri dishes and examined with the aid of a stereoscopic microscope Stemi SV6/AxioCam MRc5 (Zeiss, Oberkochen, Germany). Ciliates were collected with Pasteur micropipettes for morphological identification, ultrastructural analysis, and transcriptome sequencing.

### Transmission electron microscopy

*Balantidium polyvacuolum* specimens were fixed with 2.5% glutaraldehyde. Post-fixation was performed in 1% (v/v) osmium tetroxide in phosphate buffer solution (PBS) for 2 h at 4℃, followed by dehydration in a gradient acetone series and embedded in Araldite. Ultrathin sections were then cut on a Leica Ultracut R ultramicrotome (Leica, Germany) and stained with uranyl acetate and lead citrate. The samples were viewed using a JEM-1230 Transmission Electron Microscope (JEOL, Japan).

### Single-cell transcriptome amplification and sequencing

Single-cell samples, each comprising an individual *B. polyvacuolum* cell, were collected, lysed, and amplified to generate cDNA, according to the Smart-Seq2 protocol [18] and methods used in a previous study [19]. Qualified cDNA libraries were then loaded on the Illumina Hiseq platform for PE150 sequencing (Illumina, CA, USA).

### Transcriptome assembly and annotation

The raw Illumina reads were deposited in the Sequence Read Archive of GenBank under the accession number SRR24608340. The sequencing data were filtered by Trimmomatic v0.39 [20] in PE mode with the setting ‘ILLUMINACLIP:TruSeq3-PE.fa:2:30:10:8:true LEADING:3 TRAILING:3 SLIDINGWINDOW:4:15 MINLEN:120’, assembled using Trinity v2.13.2 [21] with the setting ‘--seqType fq --left R1.fq --right R2.fq --CPU 6 --max_memory 20G’, and decontaminated using BLAST v2.14.0 [22]. For the decontamination, the assembled transcriptome was first used as a query in a similarity search against the NCBI non-redundant (NR) database with an e-value of 1e-5 and a query-gencode of 6. Then any sequences with bacterial hits were removed from the transcriptome. Redundant sequences were removed using CD-HIT v4.6.8 [23]. The completeness of assembly was assessed using BUSCO v5.2.2 [24] against the alveolate_odb10 dataset.

Open-reading frames (ORFs) were predicted with Transdecoder v5.5.0 (http:transdecoder.github.io). To further maximize sensitivity for capturing ORFs that may have functional significance, BLASTP searches against the Swiss-Prot, Pfam and NCBI NR protein databases were conducted. The unigenes were also annotated based on the COG database. A KEGG pathway enrichment analysis was conducted with the help of EggNOG-mapper (http://eggnog-mapper.embl.de) (accessed on 1st January 2023) [25] and TBtools v1.09 [26]. Diamond v2.0.12 [27] was used for the BLAST search. Carbohydrate-active enzyme genes of *B. polyvacuolum* were identified using a hidden Markov model (HMM) search implemented in dbCAN HMMs 6.0 [28] with default parameters. The search results were then annotated against the CAZy database [29] using BLASTP. SignalP 5.0 was used to predict the protein peptide pre-sequences of these enzymes [30].

### Identification of hydrogenosomal proteins

Hydrogenosomal proteins were predicted with methods relying on the homologs of mitochondrial proteomes and methods predicting N-terminal signal peptides. First, putative hydrogenosomal proteins were detected using BLAST. The decontaminated transcriptome was used to query BLASTP, searching for similarity against a database comprising the well-described homologs of mitochondrial proteins of the ciliate *Tetrahymena thermophila* [31] and anaerobic ciliates, the latter including *C. porcatum*, *M. contortus*, *M. laminarius*, *P. frontata* and *Plagiopyla* cf. *narasimhamurtii* [14, 32] (see Additional file1-Table S5 for the sequences). Second, the identified sequences were used as queries in similarity searches against the NR database to confirm homology with other eukaryotes and to eliminate likely bacterial contaminants. All the BLAST searches used the default parameters, except for the e-value (which was 1e-5) and the query-gencode (which was 6). Third, TargetP [33] and MitoFates [34] and DeepLoc-2.0 [35] were used to predict mitochondrial-targeting signals. Finally, hydrogenosomal metabolic pathways were depicted using Adobe Illustrator.

## Results

### Transmission electron microscopy

**Fig. 1:**
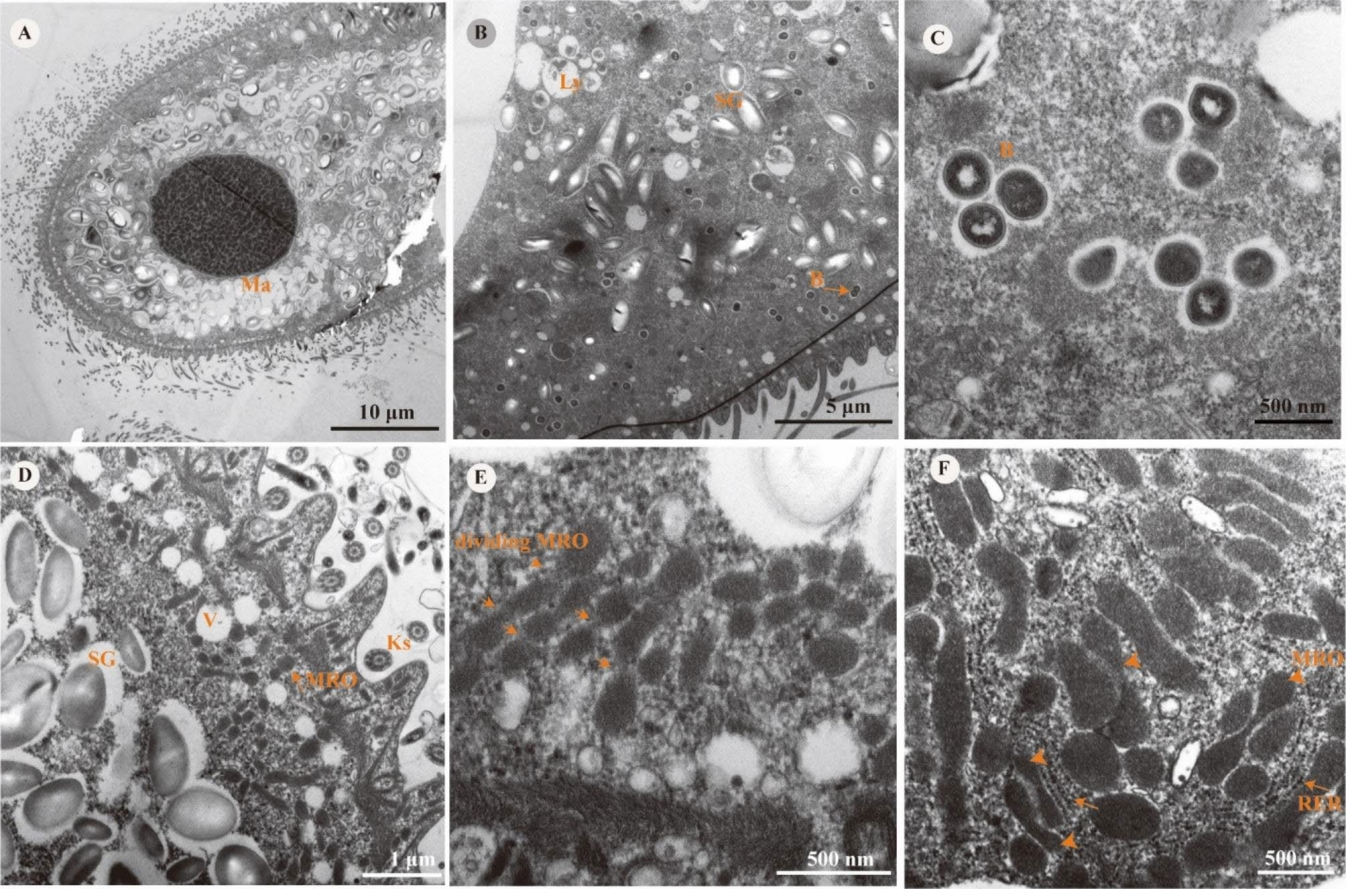
Transmission electron micrographs of *B. polyvacuolum*. **(A)** The whole body of *(B) polyvacuolum*, with macronucleus (Ma) visible. (**B)** The distribution of starch granules (SG), a lysosome (Ly) and endosymbiotic bacteria (B), with the arrow showing dividing bacteria. **(C)** A magnification of bacteria, showing the cell wall and nucleoid. **(D)** The positional relationship between starch granules (SG), mitochondrion-related organelles (MRO), vacuoles (V) and kinetosomes (Ks). **(E)** Different shapes of dividing MROs. **(F)** Arrows show the rough endoplasmic reticulum (RER) around the MROs and arrowheads show the dumbbell-shaped MROs

Transmission electron micrographs of the body of *B. polyvacuolum* (Fig. 1A) showed the presence of many starch granules and a few lysosomes, randomly distributed within the cell. Endosymbiotic bacteria were also observed in the cytoplasm (Fig. 1B). The cell membrane and the nucleoid of the endosymbiotic bacteria could also be clearly seen (Fig. 1C). Numerous mitochondrion-related organelles (MROs) were distributed in the vicinity of the starch granules (SGs), close to the cell membrane. Vacuoles of varying sizes were distributed around the MROs and kinetosomes could also be seen (Fig. 1D). The dividing MROs looked like a string of beads (Fig. 1E). Numerous rough endoplasmic reticula were located around the dumbbell-shaped and oval-shaped MROs (Fig. 1F).

### Transcriptome assembly

We obtained 12,660,018 clean reads. The number of assembled contigs was 46,639, their total length was 32,465,220 bp, and their average GC content was 38%. The average contig length was 696 bp and the maximum length was 14,666 bp. The completeness of the assembly was 58.5% based on the BUSCO assessment.

### COG annotation

In the COG annotation, 6,372 unigenes (Additional file 1-Table S1) were divided into 25 functional groups (Fig. S1). ‘Signal transduction mechanisms’ was the largest group. A total of 998 unigenes were assigned to the ‘metabolism’ category (Fig. S1). Within this category, ‘Carbohydrate transport and metabolism’, ‘Energy production and conversion’, and ‘Lipid transport and metabolism’ were the most abundant subcategories.

### KEGG annotation

The KEGG annotation results showed that 2,106 unigenes (Additional file 1-Table S2) were matched to the five functional categories of KEGG pathways (Fig S2): metabolism, genetic information processing, cellular processes, environmental information processing, and human diseases. Within the ‘Metabolism’ category, carbohydrate metabolism, energy metabolism, lipid metabolism, amino acid metabolism, and nucleotide phosphorylation were the top five annotated subcategories (Fig. S2).

### CAZymes identification

**Fig. 2:**
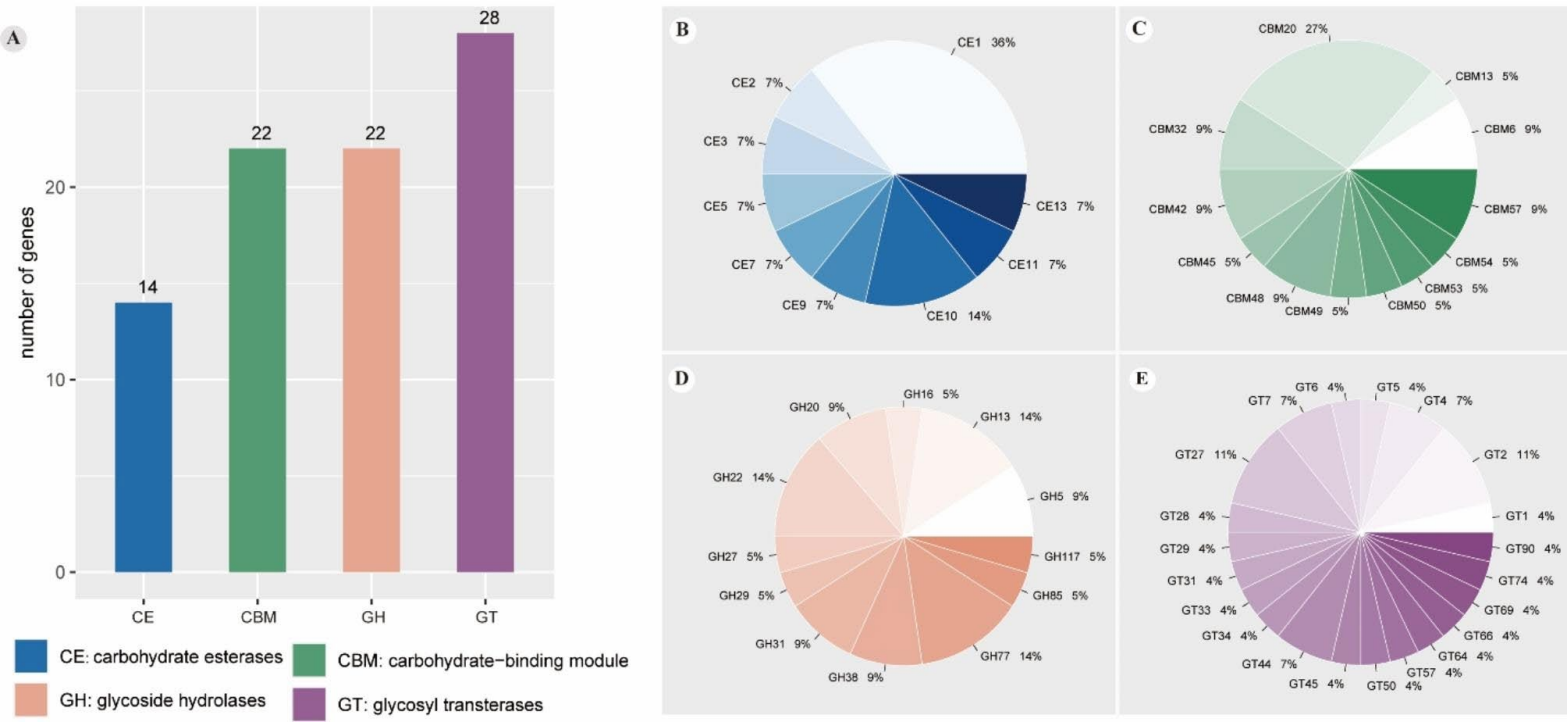
Carbohydrate-active enzymes identified in *B. polyvacuolum.***(A)** Four annotated carbohydrate hydrolase families **(B)** The type and proportion of annotated CEs. **(C)** The type and proportion of annotated CBMs. **(D)** The type and proportion of annotated GHs. **(E)** The type and proportion of annotated GTs

To understand the mechanism by which *B. polyvacuolum* contributes to the digestion of plant food in fish intestines, CAZymes were identified (Additional file 1- Table S3). Only 86 candidate CAZymes were identified in total (Fig. 2A), including 14 carbohydrate esterases (CEs), 22 with a carbohydrate-binding module (CBM), 22 glycoside hydrolases (GHs), and 28 glycosyltransferases (GTs). Among the identified CEs, CE1 accounted for the largest proportion (Fig. 2B). Among the identified CBMs, CBM20 accounted for 27% (Fig. 2C). The function of CBM20 is to bind to starch. Among the identified GHs, the proportions of GH13, GH22, and GH77 were the same as each other (Fig. 2D). The GH13 family is the major glycoside hydrolase family acting on substrates containing α-glucoside linkages. The GH5 and GH13 families act in cellulose degradation. The GH5 family can hydrolyze cellulose independently. Various GTs were also identified in this study (Fig. 2E).

In addition to the CAZymes, we also sought other evidence to show that *B. polyvacuolum* may help with plant biomass digestion. First, we used TEM for the detection of vacuoles with plant materials in *B. polyvacuolum*, which could indicate that the ciliate directly ingests plant biomass by phagocytosis. However, no such images were found, so we speculated that *B. polyvacuolum* may secrete enzymes outside in the gut environment to help with the digestion. We therefore investigated the presence of signal peptide pre-sequences on these CAZymes and found that some of them (CBM50, CBM57 and GH5) did indeed possess the signal peptide pre-sequences. As these may act in the utilization and degradation of carbohydrates such as cellulose and chitin, this suggests that *B. polyvacuolum* helps its host digest plant biomass by secreting enzymes.

### Hydrogenosome energy metabolism reconstruction

**Fig. 3:**
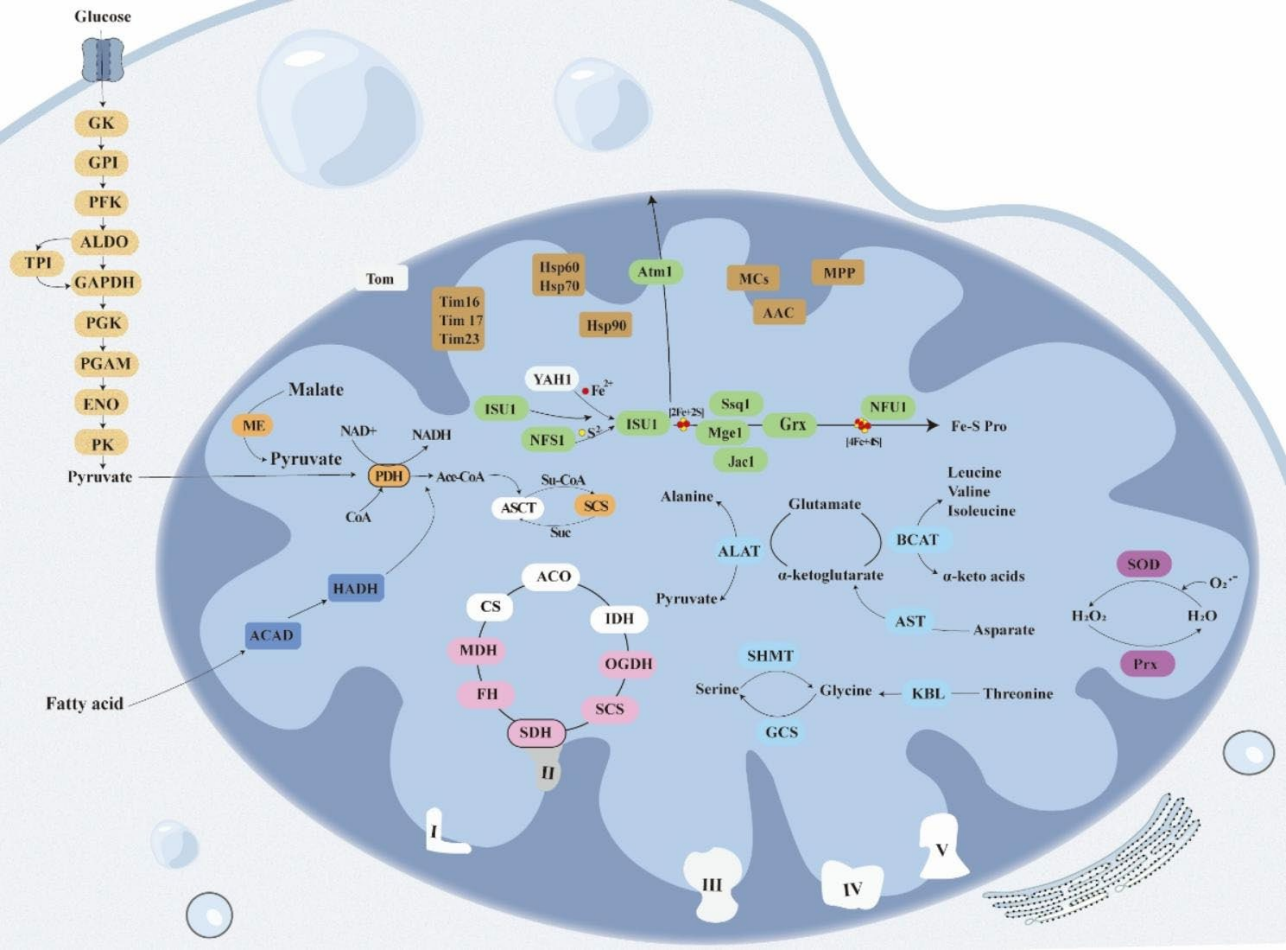
The predicted metabolic pathways of *B. polyvacuolum.* See Supplementary Table S4 for the full protein names. Proteins involved in different pathways are represented by different colors: yellow, glycolysis; pink, tricarboxylic acid (TCA) cycle; grey, electron transport chain (ETC); light-blue, amino acid metabolism; orange, pyruvate metabolism; dark-blue, fatty acid metabolism; green, iron–sulfur cluster (ISC) biosynthesis; purple, superoxide catalytic reaction; brown, other. The white shapes indicate that the protein is absent and the outlined circles indicate that some subunits of the protein have not been identified

Multiple metabolic pathways of *B. polyvacuolum* were identified based on the homologous comparison in this study, including glycolysis, pyruvate metabolism, tricarboxylic acid (TCA) cycle, amino acid metabolism, and iron-sulfur cluster (ISC) biosynthesis (Fig. 3). All enzymes involved in glycolysis were identified. Pyruvate dehydrogenase (PDH), which is responsible for the oxidation of pyruvate, was also identified in MROs. The results showed that *B. polyvacuolum* had an incomplete TCA cycle. The electron transport chain (ETC) complexes I, III, and IV and V were completely absent. Only succinate dehydrogenase subunit A (SDHA) of complex II was identified.

Amino acid metabolism-related enzymes, including alanine aminotransferase (ALAT), branched-chain amino acid aminotransferase (BCAT), aspartate aminotransferase (AST), serine hydroxymethyl transferase (SHMT), glycine cleavage system protein (GCS), and 2-amino-3-ketobutyrate CoA ligase (KBL) were identified. Among the enzymes associated with the fatty acid metabolic pathways, acyl-CoA dehydrogenase (ACAD) and 3-hydroxyacyl-CoA dehydrogenase (HADH), involved in fatty acid β-oxidation, were identified.

Several enzymes and other proteins associated with the ISC biosynthesis system of *B. polyvacuolum* were identified. Among these, cysteine desulfurase (NFS1) could be the donor of sulfur and the Fe-S cluster could first be synthesized on the iron-sulfur cluster assembly protein 1 (ISU1). Iron–sulfur cluster biogenesis chaperone (Ssq1), GrpE protein (Mge1), and Jac1 could be responsible for the transfer of [2Fe-2S] clusters within the ISC biosynthesis pathway. Meanwhile, iron-sulfur cluster scaffold protein (Nfu1) could play a role in the assembly and transport of the [4Fe-4S] cluster. The Fe^2+^ donor YAH1 was not identified.

Other enzymes involved in MRO pathways were also found, including the translocase of the inner mitochondrial membrane (Tim) complex, heat shock proteins (Hsp), ADP/ATP translocase (AAC), and the Mitochondrial Carrier Family Proteins (MCs). However, we did not detect the translocase of the outer mitochondrial membrane (Tom) complex. Superoxide dismutase (SOD) and peroxiredoxin (Prx), both of which are involved in the oxidative stress pathway, were also detected.

## Discussion

### *B. polyvacuolum* may help host fish degrade plants and algae

Xenocyprinae fishes, the hosts of *B. polyvacuolum*, are omnivorous, feeding mainly on decaying sediment, as well as diatoms, attached algae and plant material [36]. However, cellulose, as the main component of the plant cell walls, cannot be directly digested and utilized by fish [37]. Thus, help from gut microorganisms including archaea, bacteria, and protozoa is particularly important. Previous studies have mostly focused on the beneficial effects of fish gut bacteria on nutrient metabolism, immune function, and health maintenance [37–39], but little is known about the role of protozoa in the fish gut. Ciliates have been extensively studied in domestic ruminants, and research has shown that these microorganisms may play a crucial role in the degradation of plant biomass, utilization of cellulose, and energy metabolism [40–43]. On this basis, we hypothesized that ciliates may play similar roles in fish.

In indirect support of this hypothesis, we discovered many CAZymes in *B. polyvacuolum* through analysis of single-cell transcriptome data. Research has demonstrated that CAZymes act in the saccharification of polymeric carbohydrate resources, such as starch, cellulose or hemicellulose, by breaking them down into monosaccharides and oligosaccharides, which can be utilized by organisms [44]. In this study, both COG annotation and KEGG pathway enrichment results showed that *B. polyvacuolum* produces many enzymes related to carbohydrate transport and metabolism and to energy metabolism. More specifically, we identified several enzyme families putatively associated with the metabolism of complex carbohydrates. The CE1 family is one of the largest and most diverse CE families, primarily responsible for the degradation of xylan [45, 46]. The CBM20 family comprises starch-binding domains and can disrupt the helical structure of amylose [47]. The GH77 family contains starch-degrading enzymes, and it has also been identified in the rumen microbiome [48].

The presence of signal peptide pre-sequences suggests that *B. polyvacuolum* helps its host digest plant biomass by secreting enzymes. We also found a great number of SGs inside *B. polyvacuolum* and detected some glucose metabolism-related enzymes, which also indicates that *B. polyvacuolum* has the ability to degrade SGs.

Based on these findings, we hypothesize that a mutually beneficial symbiotic relationship may exist between the host fish and *B. polyvacuolum*. On the one hand, Xenocyprinae hosts provide *B. polyvacuolum* with underutilized food, a living environment, and protection from predators. On the other hand, *B. polyvacuolum* helps the host digest polysaccharides and improve the efficiency of nutrient use. In addition to *B. polyvacuolum*, however, there are also gut bacteria, fungi, and other protozoa living in the complex gut microhabitat, and the roles and interactions of these microorganisms remain unknown. More studies are therefore needed to investigate the relationship between these organisms and *B. polyvacuolum*.

### *B. polyvacuolum* uses specialized MROs to adapt to the anaerobic environment of the digestive tract

Intestinal parasitic protists have evolved diverse MROs to survive in anaerobic or microaerophilic environments. For example, *Nyctotherus ovalis* living in the intestines of cockroaches has numerous hydrogen-producing mitochondria, which have both hydrogenosome-like characteristics and canonical mitochondrion-like characteristics [11, 49, 50]. The MROs of the stramenopile *Blastocystis hominis* possess cristae, a mitochondrial genome, a TCA cycle and an incomplete ETC [8]. Recent studies of rumen ciliates, such as the entodiniomorphids *Entodinium furca* and *E. caudatum*, and the vestibuliferid *Isotricha intestinalis*, have shown that their MROs have a partial ETC and can produce ATP via substrate phosphorylation [51]. However, genomic data, both nuclear and MRO, for fish intestinal ciliates remain sparse. Thus, in this study, we tried to characterize the MROs of *B. polyvacuolum* from Xenocyprinae fish in order to contribute to a better understanding of the anaerobic adaptation of obligate endoparasites.

We found that the MROs of *B. polyvacuolum* lack almost all of the ETC complexes, except the SDHA of complex II. Similarly, only the SDHA of complex II has been identified in the anaerobic protozoa *C. porcatum* and *P.* cf. *narasimhamurtii* [14, 32], and it is possible that the SDHA can act in reverse as a fumarate reductase [32]. This suggests that a partial ETC could also possess functionality in the MROs of *B. polyvacuolum*.

Half of the TCA cycle-related enzymes from the canonical mitochondrial metabolisms were identified, which suggests that the MROs of *B. polyvacuolum* may possess canonical mitochondrial functions. Studies have shown that an incomplete TCA cycle can also run in reverse. When there are no complexes III and IV, fumarate can be converted into succinate by using electrons from rhodoquinol in anaerobic eukaryotes [52]. The presence of a homologue of the putative rhodoquinol synthesis enzyme RquA may be a clue to the presence of rhodoquinol, but more experiments need to be done to validate this.

Our results have also provided some insights into the pyruvate metabolism of *B. polyvacuolum*. Although we did not identify Fe-hydrogenase, we did identify malic enzyme. This enzyme produces pyruvate for subsequent substrate-level phosphorylation, which suggests that the MROs of *B. polyvacuolum* may be able to generate ATP via substrate-level phosphorylation, in a similar way to other intestinal anaerobic ciliates [49]. The canonical mitochondrial metabolism-related protein, the pyruvate dehydrogenase complex, is normally composed of three subunits (E1, E2, E3), but only one subunit (PDH-E1) was identified in *B. polyvacuolum* in this study. Evidence has shown that ciliates of the Spirotrichea, Armophorea, and Litostomatea (SAL) group likely use PDH, instead of the typical hydrogenosomal pyruvate:ferredoxin oxidoreductase (PFO) or pyruvate:NADP + oxidoreductase (PNO) [12, 14]. Acetyl CoA, the catalytic product of PDH, is then converted to succinyl-CoA (Su-CoA) by acetate:succinate CoA transferase (ASCT), and succinyl-CoA synthetase (SCS) then generates ATP by substrate level phosphorylation. Succinyl-CoA synthetase has been detected in this study, but not ASCT, and the alternative enzyme acetyl-CoA synthase was not detected either. This may be due to single-cell omics amplification bias, but more molecular experiments are needed in the future to validate this.

Based on the above findings, we assume that the MROs of *B. polyvacuolum* are hydrogenosomes with incomplete TCA cycles and a partial ETC complex. We plan to conduct further genomic analyses in the future, as well as studying the H_2_ production, to verify this speculation.

In addition to identifying proteins related to the TCA cycle, the ETC, and pyruvate metabolism, we also identified proteins related to the ISC system in the genome of *B. polyvacuolum*. The biogenesis of cellular iron–sulfur proteins is an essential function of mitochondria and Fe-S cluster biogenesis pathways follow three main steps: de novo synthesis of a [2Fe-2S] cluster, trafficking of the cluster and insertion into [2Fe-2S] target apoproteins, and catalytic conversion of the [2Fe-2S] into a [4Fe-4S] cluster [53]. The Fe-S cluster biogenesis pathways are highly conserved, with the ISC system operating in mitochondria, the CIA (Cytosolic Iron–sulfur protein Assembly) pathway operating in the cytosol and nucleus, and the SUF (SUlFur mobilization) system operating in plastid-containing organisms [54]. In the parasitic protists, the hydrogenosomes of *Trichomonas vaginalis* can synthesize the Fe-S cluster via the ISC system [55]. The mitosomes of *Giardia* contain components of the ISC system but lack YAH1 [56], and *Blastocystis* also contains a functional ISC system [57]. In a study on *N. ovalis*, Hsp70 and type I [2Fe-2S] ferredoxin were the only proteins involved in the Fe-S cluster to be found [49]. In this study, ISC biosynthesis system-related proteins, such as NFS1, ISU1, Ssq1, Mge1, Nfu1 and Hsp70, were detected. The only exception was YAH1, the donor of Fe^2+^. We therefore hypothesize that ISC biosynthesis may be functional in *B. polyvacuolum*.

## Conclusion

In conclusion, the metabolic characteristics of *B. polyvacuolum* were studied. This ciliate may use specialized MROs (with an incomplete TCA cycle and a large part of the ETC missing) to adapt to the anaerobic digestive tract environment. Meanwhile, it may help the host fish with degradation of plant and algal material.

### Electronic supplementary material

Below is the link to the electronic supplementary material.

Supplementary Material 1

Supplementary Material 2

## Data Availability

The raw Illumina sequences obtained during the current study are available in the Sequence Read Archive of GenBank under accession number SRR24608340 (https://www.ncbi.nlm.nih.gov/Traces/index.html?view=run_browser&acc=SRR24608340&display=metadata).

## Abbreviations

MRO: mitochondrion-related organelle
TEM: transmission electron microscopy
ORF: Open-reading frame
COG: Cluster of Orthologous Genes
ATP: adenosine triphosphate
COG: Orthologous Group
KEGG: Kyoto Encyclopedia of Genes and Genomes
PBS: phosphate buffer solution
NR: non-redundant
HMM: hidden Markov model
SGs: starch granules
CAZyme: carbohydrate-active enzyme
CE: carbohydrate esterase
CBM: carbohydrate-binding module
GH: glycoside hydrolase
GT: glycosyltransferase
TCA: tricarboxylic acid
ETC: electron transport chain
ISC: iron–sulfur cluster
ALAT: alanine aminotransferase
BCAT: branched-chain amino acid aminotransferase
AST: aspartate aminotransferase
SHMT: serine hydroxymethyl transferase
GCS: glycine cleavage system protein
KBL: 2-amino-3-ketobutyrate CoA ligase
ACAD: acyl-CoA dehydrogenase
HADH: 3-hydroxyacyl-CoA dehydrogenase
NFS1: cysteine desulfurase
ISU1: iron–sulfur cluster assembly protein 1
Ssq1: Iron–sulfur cluster biogenesis chaperone
Mge1: GrpE protein
Nfu1: iron–sulfur cluster scaffold protein
Tim: translocase of the inner mitochondrial membrane
Hsp: heat shock proteins
AAC: ADP/ATP translocase
MCs: Mitochondrial Carrier Family Proteins
Tom: translocase of the outer mitochondrial membrane
SOD: Superoxide dismutase
Prx: peroxiredoxin
CIA: Cytosolic Iron–sulfur protein Assembly
