# Supplementary material for: The energy metabolism of Balantidium polyvacuolum inhabiting the hindgut of Xenocypris davidi
Source: BMC Genomics. 2023 Oct 19;24:624. doi: 10.1186/s12864-023-09706-6 (PMC10588222; doi:10.1186/s12864-023-09706-6)
Supplement: Supplementary file 1 — Supplementary Material 1 [file 12864_2023_9706_MOESM1_ESM.docx]

**
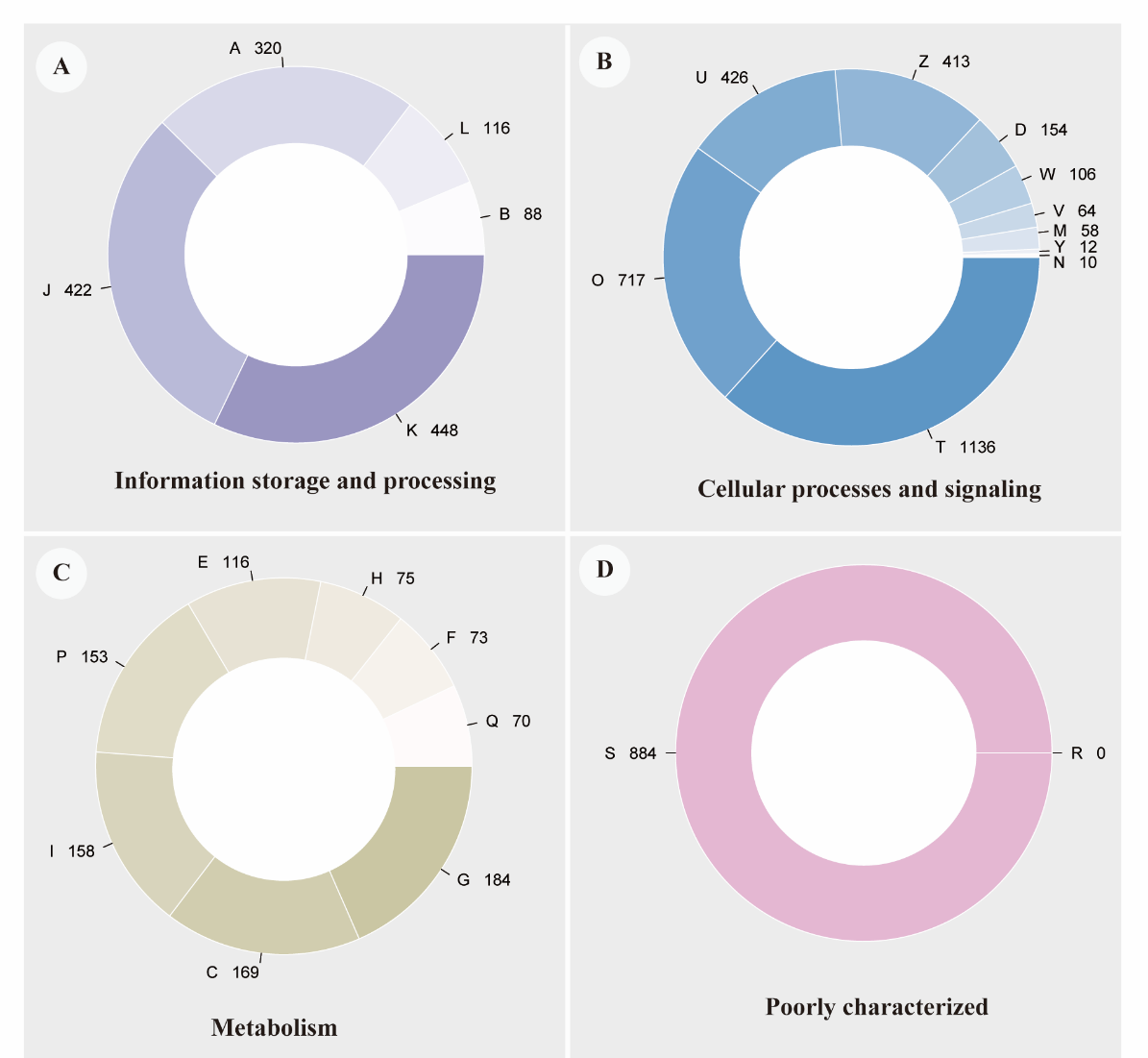
**

**Fig. S1** COG annotation of *B. polyvacuolum.* **A.** Distribution of annotated unigenes in Information storage and processing. B Chromatin structure and dynamics, L Replication, recombination and repair, A RNA processing and modification, J Translation, ribosomal structure and biogenesis, K Transcription **B.** Distribution of annotated unigenes in Cellular processes and signaling. N Cell motility, Y Nuclear structure, M Cell wall/membrane/envelope biogenesis, V Defense mechanisms, W Extracellular structures, Z Cytoskeleton, U Intracellular trafficking, secretion, and vesicular transport, O Posttranslational modification, protein turnover, chaperones, T Signal transduction mechanisms, D Cell cycle control, cell division, chromosome partitioning **C.** Distribution of annotated unigenes in Metabolism. Q Secondary metabolites biosynthesis, transport and catabolism, F Nucleotide transport and metabolism, H Coenzyme transport and metabolism, E Amino acid transport and metabolism, P Inorganic ion transport and metabolism, I Lipid transport and metabolism, C Energy production and conversion, G Carbohydrate transport and metabolism **D.** Distribution of annotated unigenes in Poorly characterized. R General function prediction only, S Function unknown.


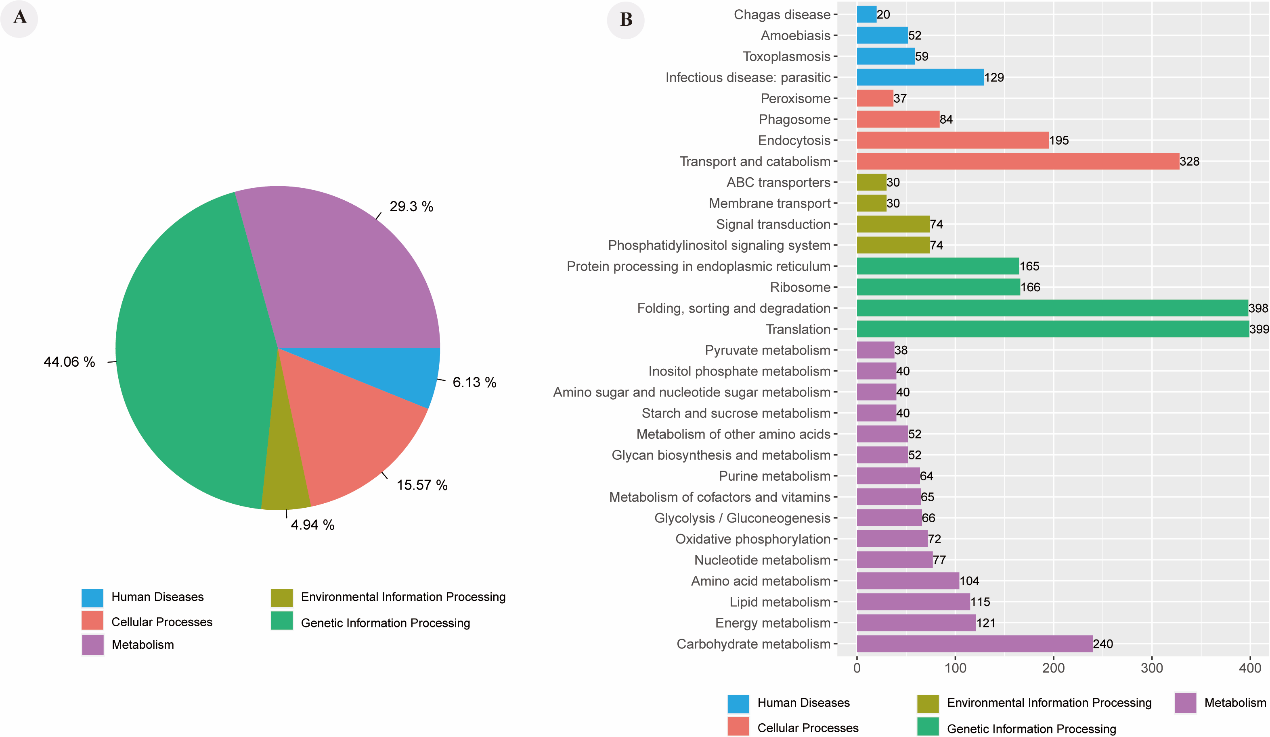


**Fig. S2** The KEGG annotation of *B. polyvacuolum* transcriptome at level_1 (**A.** overall) and level_2 (**B**. Different sub-categories).
